# Modulation of the Microenvironment Surrounding the Active Site of Penicillin G Acylase Immobilized on Acrylic Carriers Improves the Enzymatic Synthesis of Cephalosporins

**DOI:** 10.3390/molecules181114349

**Published:** 2013-11-20

**Authors:** Paolo Bonomi, Teodora Bavaro, Immacolata Serra, Auro Tagliani, Marco Terreni, Daniela Ubiali

**Affiliations:** Dipartimento di Scienze del Farmaco and Italian Biocatalysis Center, Università degli Studi di Pavia, Via Taramelli 12, Pavia 27100, Italy; E-Mails: paolo.bonomi@utc.fr (P.B.); teodora.bavaro@unipv.it (T.B.); immacolata.serra@unipv.it (I.S.); auro@ibiocat.eu (A.T.)

**Keywords:** penicillin G acylase, immobilization, microenvironment, epoxy acrylic carriers, glutaraldehyde, hydrophilization

## Abstract

The catalytic properties of penicillin G acylase (PGA) from *Escherichia coli* in kinetically controlled synthesis of β-lactam antibiotics are negatively affected upon immobilization on hydrophobic acrylic carriers. Two strategies have been here pursued to improve the synthetic performance of PGA immobilized on epoxy-activated acrylic carriers. First, an aldehyde-based spacer was inserted on the carrier surface by glutaraldehyde activation (immobilization yield = 50%). The resulting 3-fold higher synthesis/hydrolysis ratio (vs/vh_1_ = 9.7 ± 0.7 and 10.9 ± 0.7 for Eupergit^®^ C and Sepabeads^®^ EC-EP, respectively) with respect to the unmodified support (vs/vh_1_ = 3.3 ± 0.4) was ascribed to a facilitated diffusion of substrates and products as a result of the increased distance between the enzyme and the carrier surface. A second series of catalysts was prepared by direct immobilization of PGA on epoxy-activated acrylic carriers (Eupergit^®^ C), followed by quenching of oxiranes not involved in the binding with the protein with different nucleophiles (amino acids, amines, amino alcohols, thiols and amino thiols). In most cases, this derivatization increased the synthesis/hydrolysis ratio with respect to the non derivatized carrier. Particularly, post-immobilization treatment with cysteine resulted in about 2.5-fold higher vs/vh_1_ compared to the untreated biocatalyst, although the immobilization yield decreased from 70% (untreated Eupergit^®^ C) to 20%. Glutaraldehyde- and cysteine-treated Eupergit^®^ C catalyzed the synthesis of cefazolin in 88% (±0.9) and 87% (±1.6) conversion, respectively, whereas untreated Eupergit^®^ C afforded this antibiotic in 79% (±1.2) conversion.

## 1. Introduction

Penicillin G acylase (PGA, E.C. 3.5.1.11) from *E. coli* is a N-terminal serine hydrolase mainly used in the industrial production of 6-aminopenicillanic acid (6-APA) and 7-aminodesacetoxy-cephaloporanic acid (7-ADCA) for the synthesis of β-lactam antibiotics [1].

Different strategies have been developed to enhance the properties of biocatalysts (e.g., stability), including multimeric enzymes, to tailor them also for an industrial application [2,3]. Covalently immobilized PGA on solid supports results in a more stable biocatalyst, which can be easily recovered and reused [4,5,6,7,8,9,10]. However, immobilization generally exerts a detrimental effect on the synthetic performance of PGA in the kinetically controlled acylation of β-lactam nuclei, particularly when epoxy acrylic carriers such as Eupergit^®^ C or Sepabeads^®^ EC-EP are used. More hydrophilic carriers such as glyoxyl agarose have been found superior because they generate a microenvironment more favorable to the diffusion of the β-lactam nucleus into the active site [11,12]. 

Immobilization on glyoxyl agarose may be considered moderately complex at industrial scale [12] since it is a multi-step procedure and it requires reagents and conditions which are plagued by difficult handling and hazard issues, especially at a large scale (glycidol, sodium periodate, sodium borohydride, alkaline pH). Conversely, for industrial purposes, commercial epoxy acrylic supports appear to be almost ideal matrices since enzyme immobilization relies on a milder and more straightforward procedure (covalent immobilization occurs directly between nucleophiles, mainly Lys residues of the protein surface, and the epoxy-groups of the carrier) [13]. Therefore many efforts have been addressed to improve the synthetic performances of epoxy acrylic carriers. 

Differences between glyoxyl agarose and epoxy acrylic carriers become far less striking when Eupergit^®^ C or Sepabeads^®^ EC-EP are functionalized with aldehyde groups, that is the same activation commonly carried by the agarose support [11]. However, regardless of both the nature and the activation of the support, PGA immobilization prevalently occurs through the enzyme surface bearing the active site, although via different binding areas [11,14]. It is thus evident that other factors, besides the binding chemistry and the nature of carrier, concur in determining the synthetic properties of this biocatalyst. 

Steric hindrance promoted by the proximity of the support surface may prevent substrates from accessing the catalytic site. In the case of PGA, β-lactam nuclei are bulky molecules that can hardly reach the enzyme active site if this is not exposed to the medium. When covalent immobilization occurs through a spacer between the enzyme and the support, steric problems can be frequently circumvented [13,15]. Enzyme preparations where only one or a few residues of the protein are involved in the enzyme-support covalent binding through the spacer (e.g., glutaraldehyde, dextran) do not usually suffer from drastic loss of activity since the protein tends to behave as in its native state [15,16,17]. However, the lack of effect on enzyme activity [18] (due to a limited number of covalent bonds between enzyme and support and thus to a negligible “rigidification” of the protein) frequently turns into a quite poor stability of the immobilized enzyme that is thus not suitable for industrial applications. Control of the experimental conditions and of the length and nature of the spacer are indeed key points. 

Alterations of the microenvironment in terms of charge or hydrophilicity can also exert a dramatic effect on protein properties. Co-immobilization on glyoxyl agarose of PGA and polycationic polymers have been performed to enhance the stability of the biocatalyst in organic solvents through the creation of a sort of “hydrophilic shell” [19,20,21,22,23,24]. Modification of the final enzyme preparation with hydrophilic compounds (typically amino acids) yields a fully inert support by preventing from any additional interaction between the enzyme and the matrix [6,13].

**Scheme 1:**
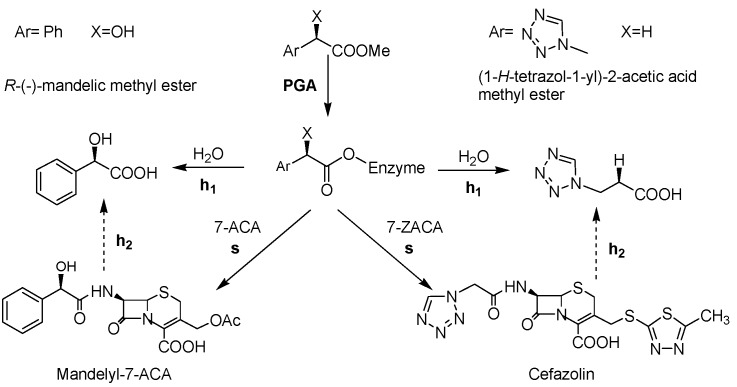
Kinetically controlled acylation of β-lactam nuclei (7-ACA and 7-ZACA, respectively) catalyzed by PGA discussed throughout the text.

*Legend:* s = synthesis; h_1_ = hydrolysis of the acyl donor [*R*-(−)-methyl mandelic ester or (1-*H*-tetrazol-1-yl)-2-acetic methyl ester, respectively]; h_2_ = secondary hydrolysis.

The catalytic properties of enzymes are affected by immobilization; this technique can be a useful tool to tune the enzyme performances [25,26]. When PGA is used in the kinetically controlled synthesis of β-lactam antibiotics (or β-lactam intermediates), immobilization can influence the synthetic performance. The synthetic efficiency of PGA is routinely assessed by determining the synthesis/hydrolysis ratio (vs/vh_1_) of the acylation of a β-lactam nucleus with an ester or an amide where *s* is the synthesis of the β-lactam antibiotic and *h_1_* is the hydrolysis of the acyl donor (Scheme 1). The highest transient yield achieved is negatively affected by *h_1 _* and by the hydrolysis of the final product (*h_2_*) [27,28]. In particular, *h_2 _* is mainly involved at the end of the synthetic process, when the concentration of the acylation product is predominant, while *h*_1_ is observable at the beginning of the reaction when the concentration of the acyl donor is high (Scheme 1). Since immobilization of PGA affects the vs/vh_1_ ratio, the evaluation of this value at the beginning of the reaction is routinely considered a suitable descriptor of the biocatalyst efficiency, in agreement with previously reported results [29,30,31].

We here described the preparation of two series of PGA-based catalysts on acrylic carriers and the application of the most promising ones in the synthesis of cefazolin (Scheme 1). In the first set of biocatalysts, a spacer was inserted on the carrier surface by glutaraldehyde activation to assess the effect of an increased distance between the enzyme and the carrier surface on the catalytic properties of PGA. The second group of biocatalysts was prepared by direct immobilization of PGA on epoxy-activated acrylic carriers, followed by quenching of unreacted oxiranes with different nucleophiles (amino acids, amines, amino alcohols, thiols and amino thiols) to assay whether a more hydrophilic microenvironment correlates with higher synthetic performances.

## 2. Results and Discussion

### 2.1. Immobilization of PGA through a Spacer

**Scheme 2:**
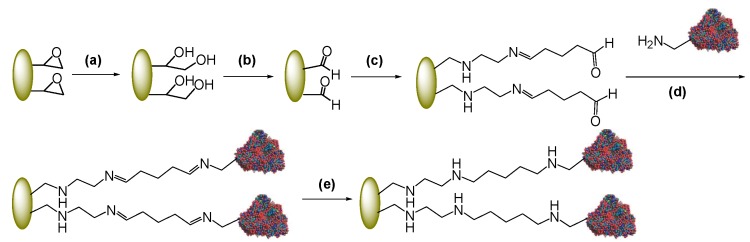
Immobilization of PGA through a amino-alkyl spacer.

Epoxy acrylic supports (Eupergit^®^ C and Sepabeads^®^ EC-EP) have been oxidized to their aldehyde forms after conversion of the epoxy groups into diols via acid hydrolysis [11] (Scheme 2a–b). To introduce a spacer between the enzyme and the surface of the carrier, aldehyde-Eupergit^®^ C and aldehyde-Sepabeads^®^ EC-EP have been reacted with ethylendiamine and then with glutaraldehyde by Schiff’s base chemistry (Scheme 2c) [32]. As a result, aldehyde groups of the amino-alkyl spacer reacting with the amino groups of the protein are located far from the surface of the matrix. Immobilization occurs, again, through the formation of Schiff’s bases generated from the reaction between the enzyme amino groups and the aldehyde groups of the spacer. Chemical reduction of imines to stable C-N bonds completes the two-step process (Scheme 2d–e) [8]. As a consequence of the activation protocol, the resulting matrix has three different functionalities: the primary amino groups, the fairly hydrophobic chain of glutaraldehyde and the aldehyde reactive groups. Depending on the immobilization conditions, it has been reported that the first event of immobilization may have different causes and involve different areas of the enzyme surface [33]. However, in all cases, this immobilization protocol increases the distance between the carrier surface and the protein. 

*Experimental conditions*: (a) 0.5 M H_2_SO_4_, r.t., 2 h, (see ref. [9]); (b) 100 mM NaIO_4_, r.t., 2 h, (see ref. [9]); (c) reaction with ethylendiamine (EDA): 2 M EDA, pH 10, r.t., 2 h; NaBH_4_, 2 h. Reaction with glutaraldehyde (GA): GA 25% v/v in H_2_O, pH 7, r.t., 16 h; (d) PGA (200 IU/g), pH 7, r.t., 3 h; (e) NaBH_4_, pH 7, 30 min.

Our goal was to produce a biocatalyst with such a high degree of freedom to mimic the enzyme in its native state, by reducing (or even overriding) any steric hindrance caused by the proximity of the support surface to the active site. For this reason, in this case, immobilization was performed at moderate ionic strength and neutral pH [12,33] to drive Schiff’s bases formation towards the terminal amino groups of the enzyme, exclusively, without involving surface amino groups (mostly Lys). The pH of the immobilization solution strongly affects the reactivity of the amino groups allowing for a sort of “selection” of the amines involved in the reaction with the support. Being the pKa value of the NH_2_ terminus around 7−8, at neutral pH this group is far more reactive than the average Lys residues (whose pKa is estimated to be around 10). Therefore, this immobilization initially results in a single amino group-support bond. Other bonds can then occur with Lys nearby the first imino bond to afford a multipoint interaction, especially when highly activated carriers are used. It is plausible that the first bond between PGA and the carrier occurs with the N-terminal-amino group of the α-chain (Glu1), as the N-terminal-amino group of the β-chain (Ser1) is buried in the enzyme’s active site. A similar scenario, where immobilization is designed to make the active site highly accessible to substrates, was achieved by site directed mutagenesis introducing a flexible tag of three Lys alternating with three Gly nearby the N-terminus [34,35].

It should not be underestimated that glutaraldehyde structure in aqueous solution is not limited to the monomeric form but also dimers, trimers, polymers, cyclic hemiacetal can occur leading to many different possible reaction mechanisms upon reaction of glutaraldehyde with proteins [36]. However, each of these structures would be expected to form Schiff’s bases upon nucleophilic attack by Lys residues in a protein that turns into an increased distance between the enzyme and the carrier [36]. Scheme 2 represents indeed a simplified sketch of immobilization of PGA through a glutaraldehyde-based spacer.

Glyoxyl agarose was activated with ethylendiamine and glutaraldehyde according to the route above described and then it was used for PGA immobilization as well. The synthetic efficiency of PGA is routinely assessed by determining the synthesis/hydrolysis ratio (vs/vh_1_), as stated in the Introduction (Scheme 1) [29,30,31,35]. This ratio depends on the affinity of the β-lactam nucleus for the enzyme active centre and describes the percentage of synthesis at the beginning of the reaction (percentage of acylating agent converted into the acylation product): a high value of vs/vh_1_ means that the β-lactam nucleus is well adsorbed in the enzyme’s active site and the acyl moiety is efficiently transferred on the β-lactam core. As the reference reaction for such an evaluation, the synthesis of mandelyl-7-ACA (from 7-aminocephaloporanic acid, *i.e.*, 7-ACA, and *R*-(−)-methyl mandelic ester) was here considered (Scheme 1).

**Figure 1:**
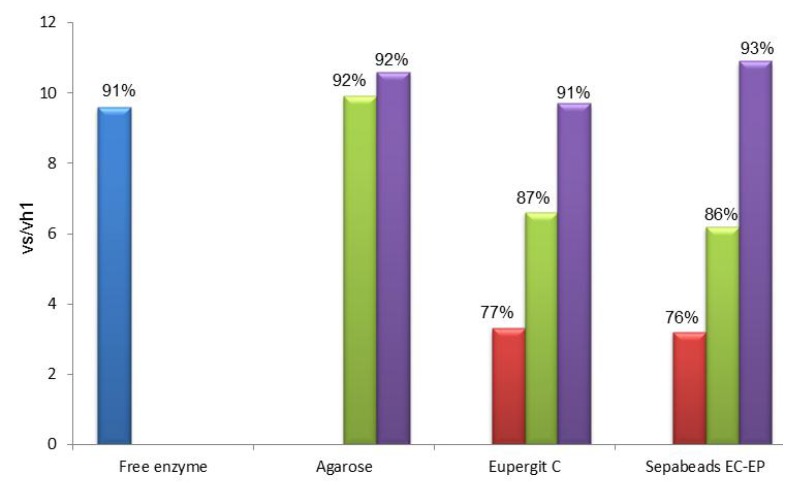
Selectivity of the PGA biocatalysts between the *N*-acylation reaction (vs) of 7-ACA and the hydrolysis of *R*-(−)-methyl mandelic ester (vh_1_).

Results obtained in the synthesis of mandelyl-7-ACA catalyzed by PGA immobilized through the amino-alkyl spacer on different carriers are depicted in Figure 1. For a comparative evaluation, both vs/vh_1_ and percentage of conversion were reported for all the prepared PGA-based catalysts (with or without the spacer) and the native enzyme.

From the inspection of Figure 1 it is evident that the presence of a spacer between the enzyme and the support produced, in all cases, an enhancement of the synthetic catalytic properties of the immobilized PGA in the studied reaction, as supported by the higher values of vs/vh_1_. Results obtained with epoxy acrylic carriers upon functionalization with aldehyde groups and subsequent ethylendiamine-glutaraldehyde activation is noteworthy: vs/vh_1_ of both acrylic carriers (9.7 ± 0.7 for Eupergit^®^ C and 10.9 ± 0.7 for Sepabeads^®^, respectively) reached the value of glyoxyl agarose (9.9 ± 1.0), which is the highest performing carrier for PGA, since its synthetic behavior is comparable to that of the native enzyme (9.6 ± 0.4). These data are consistent with a minor steric hindrance achieved through the use of the spacer that also reduces the negative effect of the hydrophobic surface by driving the catalytic site away from the carrier.

*Reaction conditions (saturating conditions)*: 4 °C, pH 6.5, acyl donor 10 mM, 7-ACA 50 mM. Data reported above each column refer to the highest percentage of conversion. Percentage of conversion was calculated as following: (vs/vs + vh_1_) × 100 (see Experimental). *Immobilization*: loading = 200 IU/g (14.3 mg of protein). *Legend*: aldehyde activation (green) (from ref. [9]); epoxy activation (red) (from ref. [9]); glutaraldeyde activation (purple).

Interestingly, if one considers the data of the set of PGA-base biocatalysts on Eupergit^®^ C or Sepabeads^®^ EC-EP it clearly appears that when the acrylic supports carry the same activation of the most favorable carrier (agarose) the vs/vh_1_ is higher than the value obtained with the epoxy activation. The effect played by the nature of the support thus emerges as individual contribution to the enzyme performance: the detrimental influence exerted by the hydrophobic matrix, although attenuated by aldehyde functionalization, can be fully quenched only by the addition of a spacer. This evidence confirms that the more exposed to the medium (and far from the hydrophobic support) is the catalytic pocket, the higher is the synthetic performance of the biocatalyst.

It is worth mentioning that no difference was observed when PGA was immobilized on glyoxyl agarose or on glutaraldehyde-activated agarose. In this case, the more hydrophilic nature of this carrier has *per sé* a positive effect and the spacer does not further enhance the catalytic properties of PGA.

Data reported in Figure 1 were obtained when a large excess of β-lactam nucleus (50 mM) was used in the PGA-catalyzed acylation (*i.e.*, in “saturating” conditions) to enhance the achievement of the highest conversion into the product. In this case the active site is “saturated” with the β-lactam nucleus which can efficiently compete with water for the acyl-enzyme complex (Scheme 1). However, even in “nonsaturating” conditions (β-lactam nucleus: 5 mM) glutaraldehyde-activated acrylic carriers showed the highest vs/vh_1_ among all the considered immobilized biocatalysts (Figure S1, Supplementary Materials).

Immobilization yields on glutaraldehyde-activated carriers were moderate to high in all cases and never below 50%.

### 2.2. Post-Immobilization Modification of Epoxy Acrylic Carriers (Eupergit^®^ C)

From the results previously discussed it is evident that the catalytic behaviour of PGA depends on several factors and, mostly important, that the interplay of immobilization techniques and experimental conditions can modulate the synthetic performance of the enzyme when it is immobilized on acrylic carriers till resembling the non immobilized enzyme. Again, the effect of the nature of the support has been confirmed to play a pivotal role [11]. 

**Scheme 3:**
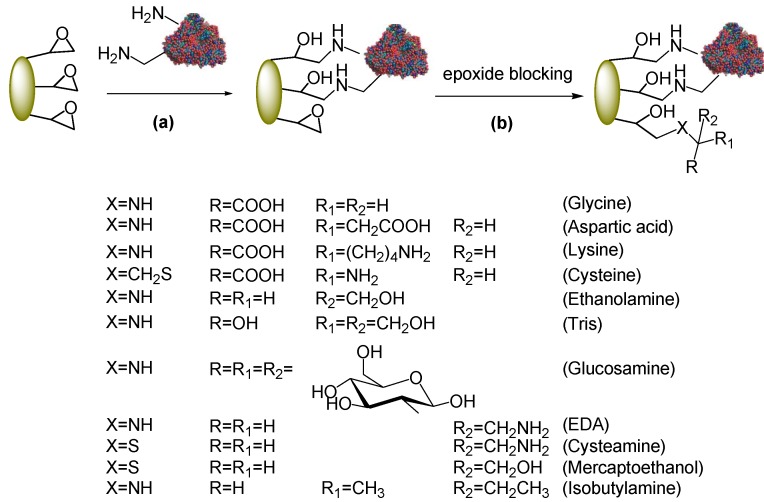
Quenching of unreacted epoxy groups after immobilization.

Being fairly hydrophobic, acrylic carriers negatively affect the catalytic properties of PGA in the synthesis of β-lactam antibiotics. A more hydrophilic (and fully inert) support can be obtained after the two-step immobilization (*i.e.*, hydrophobic adsorption and covalent attachment) by blocking the remaining epoxy groups of the carrier with hydrophilic nucleophiles (Scheme 3). This step can alter the physical properties of the support surface, quenches the enzyme-support reaction and prevents substrates from reacting with the support upon catalysis. Amino acids such as glycine are generally used to this end [13]. We here used a representative series of different nucleophiles (amino acids, amines, amino alcohols, thiols, amino thiols) to assay whether the hydrophilic/hydrophobic balance correlates with the synthetic performances. To this aim, some reagents were selected to introduce a net charge on the carrier (e.g., amino acids), others (e.g., amino alcohols) according to a polarity ranking, whereas ethylendiamine (EDA), cysteamine and mercaptoethanol were useful to compare the effect of an hydroxyl group against an amino group (for cysteamine and mercaptoethanol it is plausible that blocking of the support epoxides occurs through the SH group, being a stronger nucleophile). Isobutylamine was the negative control as this molecule concurs to increase the hydrophobic properties of the support. All reactions were performed with Eupergit^®^ C preparations at pH 9.5−10 to enhance the reactivity of the nucleophiles. Epoxy groups are very stable under alkaline conditions for long-term incubations.

*Experimental conditions*: (**a**) 1–1.5 M phosphate buffer, pH 8, 24 h; (**b**) 1–3 M blocking reagent, 50 mM NaHCO_3_ pH 10 containing 50 mM phenylacetic acid, 24 h. Blocking reagents are given in brackets.

**Figure 2:**
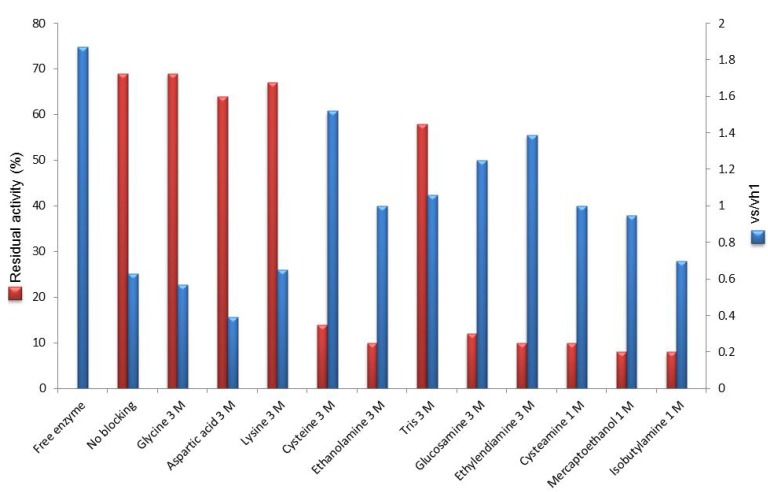
Residual activity and vs/vh_1_ of immobilized PGA on Eupergit^®^ C after post-immobilization quenching of unreacted epoxides.

According to established protocols, blocking reagents were added in very high concentration (1–3 M, depending on their solubility) [6,13] at the end of the immobilization step (24 h) and kept under stirring for further 24 h. For each blocking reagent, the residual activity of the biocatalyst after the overall process (48 h) is depicted in Figure 2.

*Reaction conditions (nonsaturating conditions)*: 4 °C, pH 6.5, substrates (acyl donor and 7-ACA) 5 mM. *Legend*: percentage of residual activity after post-immobilization blockage (red); vs/vh_1_ (blue). *Immobilization*: loading = 200 IU/g (14.3 mg of protein).

In most cases the activity of the final enzyme preparation was sensitively decreased. The series of amino acid blocking reagents appeared not to exert negative effect (glycine, aspartic acid and lysine) with the exception of cysteine. In fact, after cysteine quenching, the immobilization yield was about 15% (30 IU/g).

Surprisingly, Tris was the outlier in a quite constant trend thus showing that in the series of amino alcohols a correlation between the number of hydroxyl groups and the residual activity cannot be found.

Loss of activity of the immobilized biocatalyst upon post-immobilization treatment can be acceptable if it is compensated by a high synthetic performance. To verify this issue, all the enzyme preparations were assayed in the synthesis of mandelyl-7-ACA (Scheme 1 and Figure 2). To better highlight differences among all the tested biocatalysts, *N*-acylation of 7-ACA with *R*-(−)-methyl mandelic ester was carried out in nonsaturating concentration (β-lactam nucleus: 5 mM). Under saturating conditions (β-lactam nucleus: 50 mM), substrate diffusion to the catalytic site is maximized and differences in the synthetic performances tend to collapse (see Figure S1, Supplementary Materials).

On the contrary, nonsaturating conditions highlight the accessibility of the active site and the selectivity for the synthetic reaction (vs/vh_1_) can be better correlated, in this case, with the post-immobilization treatment.

Interestingly, if one compares the two series of data reported in Figure 2 it can be noticed that the the less enzyme activity is retained after epoxide blocking, the higher is the vs/vh_1_. Amino acids did not exert any remarkable effect on the residual activity of immobilized PGA and their selectivity for the synthetic reaction was retained, being comparable to that of the unmodified biocatalyst (the only exception is cysteine). All other blocking reagents resulted in an improvement, although to a different extent, of the vs/vh_1_. The increased hydrophilicity of the microenvironment may be a plausible explanation for the observed improvement (as supported by the trend of amino alcohols) but not exhaustive, as suggested by the value found for isobutylamine.

The most relevant result was undoubtely provided by post-immobilization modification of the biocatalyst with cysteine. In this case the vs/vh_1_ was by far higher (1.52) with respect to the untreated biocatalyst (0.63) and very close to the value measured for the non immobilized PGA (1.87). However, the very positive result of this biocatalyst in the synthesis was associated with a poor activity recovery (about 15%) upon post-immobilization epoxide quenching (Figure 2).

**Figure 3:**
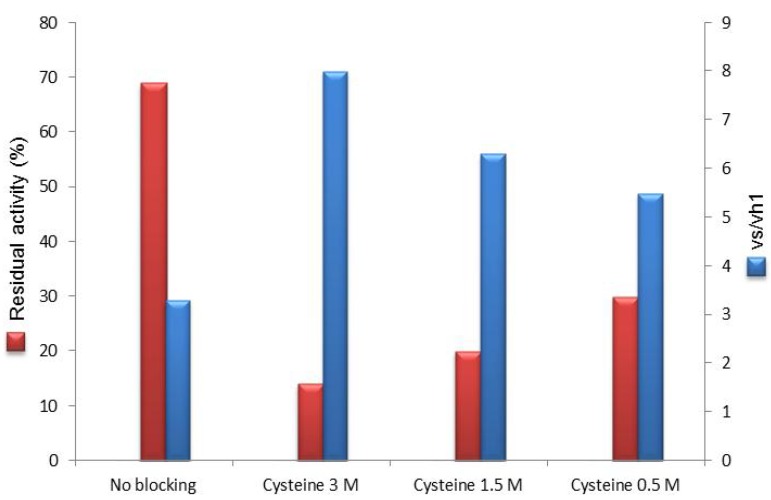
Use of cysteine (3–0.5 M) as post-immobilization epoxide blocking.

In an attempt to find a balance between residual enzyme activity after cysteine blockage and synthetic performance, we tested concentrations lower than 3 M (1.5 and 0.5 M) of this amino acid as quenching reagent. Although with 0.5 M cysteine the enzymatic residual activity was increased by 2-fold (Figure 3), this result was not associated with the same improvement in the vs/vh_1_ (vs/vh_1_ = 5.5) that was lower than the reference value (3 M cysteine). 

Percentage of enzymatic activity recovery after blockage and vs/vh_1_ are reported (red and blue, respectively). *Reaction conditions (saturating conditions):* 4 °C, pH 6.5, acyl donor 10 mM, 7-ACA 50 mM. *Immobilization*: loading = 200 IU/g (14.3 mg of protein).

Overall, 1.5 M appeared to be the optimal value to conjugate enzyme activity recovery and efficiency in the synthetic application (20% of recovered activity and vs/vh_1_ = 6.3).

**Figure 4:**
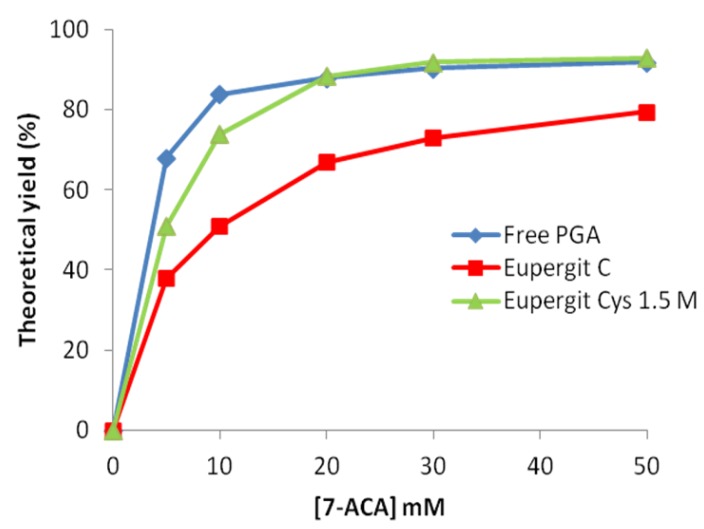
Theoretical yield of *R*-(−)-mandelyl-7-ACA at increasing concentrations (5–50 mM) of 7-ACA.

Curves depicted in Figure 4 represent the theoretical yield (%) of mandelyl-7-ACA from the acylation of 7-ACA upon increasing concentration of the β-lactam nucleus. This plot clearly shows that different enzyme preparations can perform more similarly at the beginning of the reaction (when the concentration of the β-nucleus is high), whereas as more as the reaction proceeds (and the β-lactam nucleus concentration decreases), the different ability of the examined catalysts in converting the β-lactam nucleus into the acylation product is highlighted. This comparison can be very informative in the view of preparative applications to address the choice of the biocatalyst. It is evident here that no difference can be detected between PGA immobilized on Eupergit^®^ C treated with 1.5 M cysteine and the non- immobilized enzyme in saturating conditions. PGA immobilized on untreated Eupergit^®^ C is much less performing than the cysteine-treated counterpart both in nonsaturating and in saturating conditions.

*Reaction conditions*: 4 °C, pH 6.5, *R*-(−)-methyl mandelic ester: 5 mM. Theoretical yield was calculated as following: (vs/vs + vh_1_) × 100 (see Experimental).

The difference between vs/vh_1_ of the PGA immobilized on Eupergit^®^ C (3.3) and the cystein-treated counterpart (6.3) (Figure 3) prompted us to a further investigation.

We hypothesized that the higher vs/vh_1_ of the cysteine-treated PGA preparation might be ascribed to the higher substrate (β-lactam nucleus) concentration achieved in the active site as a result of a more hydrophilic microenvironment. According to this, a drop of the rate of ester hydrolysis (vh_1_) and an enhancement of the rate of synthesis (vs) could be expected since the β-lactam nucleus can more easily “compete” with water for the acyl-enzyme complex (see Scheme 1).

**Figure 5:**
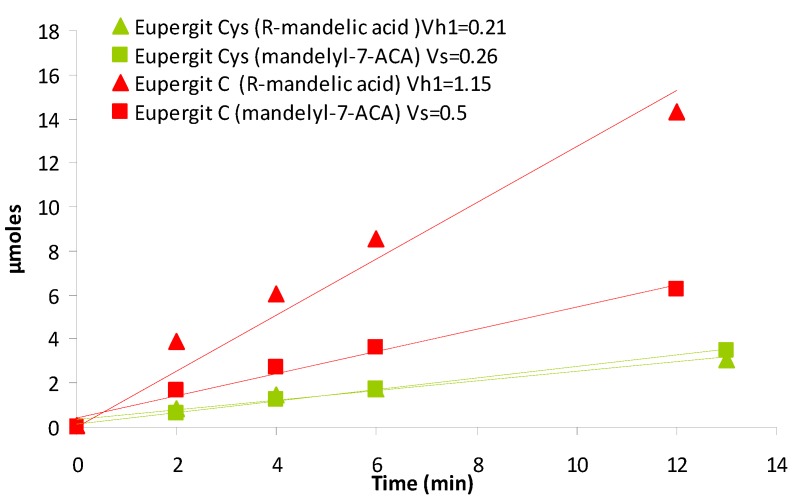
Vs (square) and vh_1_ (triangle) of the reaction catalyzed by PGA immobilized on Eupergit^®^ C before (red) and after quenching with 1.5 M cysteine (green).

To verify this, micromoles of product (mandelyl-7-ACA and mandelic acid) obtained by PGA-catalyzed bioconversion in nonsaturating conditions were plotted against time course for the PGA immobilized on Eupergit^®^ C and the cystein-treated counterpart (1.5 M) (Figure 5). 

**Figure 6:**
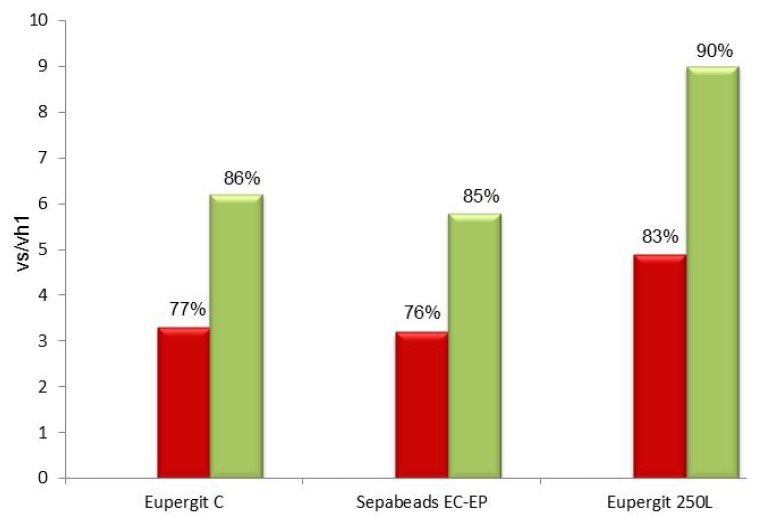
Vs/vh_1_ and percentage of conversion (synthesis of mandelyl-7-ACA) of PGA immobilized on epoxy acrylic carriers (Eupergit^®^ C, Sepabeads^®^ EC-EP and Eupergit^®^ 250L) before and after quenching with cysteine.

Post-immobilization blocking with 1.5 M cysteine was also applied to Sepabeads^®^ EC-EP and Eupergit^®^ C 250L. In both cases, vs/vh_1_ (in saturating conditions) were up to 2-fold higher than the values achieved with the untreated biocatalysts and the percentage of conversion in the synthesis of mandelyl-7-ACA was increased by about 10% (Figure 6). Sepabeads^®^ EC-EP gave the same data obtained with Eupergit^®^, whereas Eupergit^®^ C 250L was slightly superior because of its larger pore [37] dimensions which results in an enhancement of mass-transfer.

*Reaction conditions (non-saturating conditions)*: 4 °C, pH 6.5, substrates (acyl donor and 7-ACA) 5 mM; immobilized PGA = 0.15 g.

*Reaction conditions (saturating conditions)*: 4 °C, pH 6.5, acyl donor 10 mM, 7-ACA 50 mM. Data reported above each column refer to the highest percentage of conversion. Percentage of conversion was calculated as following: (vs/vs + vh_1_) × 100 (see Experimental). *Legend*: epoxy activation (red) (from ref. [9]); 1.5 M cysteine quenching (green).

### 2.3. Synthesis of Cefazolin

PGA immobilized on Eupergit^®^ C blocked with 1.5 M cysteine was used in the synthesis of Cefazolin at a preparative concentration (Scheme 1). *N*-acylation was performed with a 3:1 excess of the ester to achieve the complete conversion of the β-lactam nucleus (7-ZACA, 50 mM) into the product. In this synthesis the acylation product is not hydrolyzed by the PGA from *E. coli* [11,29,30] and thus its concentration remains almost constant after reaching the highest value. For this reason, h_2_ is negligible (see Scheme 1), the percentage of conversion is only influenced by the hydrolysis of the acylating ester (vh_1_) and the yield depends exclusively on the vs/vh_1_ ratio and therefore on the catalytic behavior of the biocatalyst.

Table 1 reports the comparison of four enzyme preparations: PGA immobilized on Eupergit^®^ C as such and upon glutaraldehyde- and cysteine-activation; PGA immobilized on glyoxyl agarose was also considered as it represents the highest performing PGA-based catalyst. 

**Table 1:** Percentage of conversion obtained in the synthesis of Cefazolin catalyzed by different PGA preparations (see also Scheme 1).

Support	Activation	Time (min)	Conversion (SD%)
Eupergit^®^ C	Epoxy ^a^	180	79% (1.2)
Eupergit^®^ C	Glutaraldehyde	330	88% (0.9)
Eupergit^®^ C	“Epoxy-Cysteine”	270	87% (1.6)
Agarose	Aldehyde ^a^	330	90% (1.8)

*Experimental conditions*: 150 mM acyl donor, 50 mM β-lactam nucleus (7-ZACA), PGA: 50 IU. The reaction was initiated at pH 7.5 to ensure the complete solubilization of 7-ZACA and then maintained at 6.5 (see ref. [11]). ^a^ From ref. [11].

## 3. Experimental

### 3.1. General

The extracts of PGA from *E. coli* as well as penicillin G potassium salt (PGK), the β-lactam nuclei, the analytical standards of cefazolin and (1-*H*-tetrazol-1-yl)-2-acetic acid methyl ester were kindly provided by ACS Dobfar SpA (Tribiano, Milano, Italy). The protein concentration of PGA extract was 100 mg/mL with a specific activity towards PGK of 14 IU/mg. Cross-linked 6% agarose beads (Sepharose 6B-CL) was from Amersham Biosciences AB (Uppsala, Sweden). Sepabeads EC-EP was a gift of Resindion (Binasco, Milano, Italy), while Eupergit^®^ C was from Röhm Pharma (Darmstadt, Germany). *R*-(−)-Methyl mandelic ester, and all other reagents and solvents (HPLC grade) were purchased from Sigma-Aldrich (Milano, Italy).

The pH of the solutions during the enzymatic hydrolyses and syntheses was kept constant by using an automatic titrator 718 Stat Titrator from Metrohm (Herisau, Switzerland). HPLC analyses were run on a BIO-TEK Kontron (Milano, Italy) instrument equipped with a UV detector 535. The column was a RP_18_ Interchrom 300 × 3.9 mM (5 mM). Protein concentration was determined by the Bradford assay using bovine serum albumin as standard on a Shimadzu spectrophotometer UV 1601 (Milano, Italy).

### 3.2. Enzymatic Activity Assay

The hydrolytic activity of PGA was measured in international units (IU). One IU corresponds with the amount of enzyme that liberates one mmol of phenylacetic acid per minute from PGK, as previously reported [34].

### 3.3. Enzyme Immobilization

All enzyme derivatives were prepared by loading 14.3 mg of enzyme per gram of carrier (corresponding to about 200 IU).

#### 3.3.1. Immobilization of PGA on Epoxy Acrylic Supports

The epoxy-activated acrylic resins (Eupergit^®^ C and Sepabeads^®^ EC-EP) were used according to the supplier indications. Immobilization was carried out at high phosphate concentration (1–1.5 M), pH 8.0, for 24 h at room temperature as previously described [11].

#### 3.3.2. Immobilization of PGA on Aldehyde Supports

Preparation of aldehyde agarose and immobilization were performed according to the procedure previously described [8]. Similarly, starting from epoxy acrylic carriers, aldehyde activation and immobilization were done according to the procedure previously reported [11].

#### 3.3.3. Immobilization of PGA on Glutaraldehyde-Activated Acrylic Resins

Immobilization of PGA on supports activated with glutaraldehyde was performed according to the procedure previously described, starting from aldehyde carriers [32]. Activation and immobilization was performed in three steps:

*Step 1*. *Reaction of aldehyde activated carriers with ethylenediamine*. The aldehyde support (3 g), was suspended in 2 M ethylenediamine pH 10.0 (38.7 mL) and kept at room temperature under mechanical stirring for 2 h. Then, NaBH_4_ (43 mg, 1 mg per mL of suspension) was added and the reaction mixture was maintained under stirring for additional 2 h. The support was then filtered under reduced pressure on a glass filter, washed with distilled water and used for the activation with glutaraldehyde.

*Step 2*. *Activation with glutaraldehyde*. The aminated carrier (3 g) was suspended in 0.2 M phosphate buffer pH 7.0 (3.4 mL), then a solution of 25% (v/v) glutaraldehyde (5.1 mL) was added. The mixture was kept under mechanical stirring for 16 h at room temperature in absence of light. The resin was filtered, washed with distilled water and immediately used for enzyme immobilization.

*Step 3*. *Immobilization of PGA on carriers activated with glutaraldehyde*. The glutaraldehyde activated carrier (4 g) was suspended in 50 mM bicarbonate buffer pH 7.0 (22.8 mL) containing 100 mM phenylacetic acid. The appropriate amount of enzyme extract to have 200 IU/g was added and maintained under stirring for 3 h at room temperature. The suspension was diluted with a solution of NaBH_4_ in 50 mM bicarbonate buffer (28.5 mL, 2 mg/mL) containing 100 mM phenylacetic acid, pH was adjusted to 10.0 by adding NaOH. After 30 minutes, the enzyme was filtered, washed with 10 mM phosphate buffer pH 5.0 and subsequently with distilled water.

### 3.4. Modification of Epoxy-Activated Acrylic Carriers

The immobilized enzyme (5 g) was suspended in a solution (28.6 mL) of the suitable amount of blocking reagent dissolved in 50 mM bicarbonate buffer containing 50 mM phenylacetic acid (28.6 mL), the pH was adjusted to 10.0 with NaOH and the suspension was kept under gentle stirring for 24 h. The enzyme preparation was filtered, washed with 100 mM phosphate buffer pH 7.0 and then with distilled water.

### 3.5. Enzymatic Reactions

(a) The vs/vh_1_ ratio of the *N*-acylation of 7-ACA with *R*-(−)-methyl mandelic ester and the percentage of synthesis were evaluated by measuring the initial rate of the synthesis (vs) of the acylation product and of the rate of ester hydrolysis (vh_1_) to give *R*-mandelic acid at the beginning of the reaction (before 20% of ester was converted), according to the general procedure previously reported [34]. The reactions were monitored by HPLC analysis at 220 nm (eluent: 20% CH_3_CN in 10 mM phosphate buffer pH 3.2). The percentage of synthesis was calculated using the following equation: [vs/(vs + vh_1_)] × 100.

(b) The synthesis of cefazolin was performed by *N*-acylation of 7-ZACA with (1-*H*-tetrazol-1-yl)-2-acetic acid methyl ester, according to the procedure previously reported [11,13]. The reactions were monitored by HPLC analysis at 274 nm (eluent: 20% CH_3_CN in 10 mM phosphate buffer pH 3.2).

### 3.6. Stability of the Immobilized Preparations

Each immobilized enzyme preparation (500 mg) was added to a solution (5 mL) containing 10 mM phosphate buffer and 40% of MeOH (v/v) at pH 7.5 or 10 mM phosphate buffer at pH 7.5. The mixture was stirred at room temperature or at 50 °C, respectively, for 48 h. At fixed times, 500 µL of suspension were withdrawn and the residual activity was evaluated by standard activity assay (PGK test).

## 4. Conclusions

Acrylic carriers bearing epoxy groups such as Eupergit^®^ C and Sepabeads^®^ EC-EP rely on a straightforward procedure for enzyme immobilization. In the case of PGA, however, immobilization on those carriers negatively affects the synthetic properties of the enzyme. The introduction of a spacer between the enzyme and the support resulted in an improvement of the synthetic performance of the biocatalyst, consistently with the increased distance between the enzyme and the carrier surface. This result was in agreement with that obtained when a specific amino acid tag was introduced on the protein surface via site directed mutagenesis to generate a preferential immobilization point. However, immobilization through glutaraldehyde is a complex multi-step procedure which is hardly feasible for applicative purposes. 

Immobilization on acrylic epoxy carriers, followed by post immobilization treatment with the appropriate quenching reagent, is far easier to perform also at a large scale. Post-immobilization quenching of oxiranes by reaction with hydrophilic nucleophiles generally increased the selectivity of the enzyme for the synthesis. This result was (partly) ascribed to the generation of a more hydrophilic microenvironment which can “compensate” for the detrimental effects exerted by the hydrophobic support. However, a drop in the enzyme recovered activity was registered in some cases. Blocking of the epoxy acrylic carriers (Eupergit^®^ C, Sepabeads^®^ EC-EP and Eupergit^®^ C 250L) with cysteine resulted in high performing biocatalysts, even comparable with the non-immobilized enzyme but with the advantage of a heterogeneous catalysis.

## Abbreviations

7-ACA: 7-amino-cephalosporanic acid
EDA: ethylendiamine
GA: glutaraldehyde
PGK: penicillin G potassium salt
7-ZACA: 7-amino-3-(5-methyl-1,3,4-thiadiazol-2-yl)thiomethyl-3-cephem-4-carboxylic acid

## Supplementary Materials

Supplementary materials can be accessed at: http://www.mdpi.com/1420-3049/18/11/14349/s1.

## References

[1] Chandel A.K., Rao L.V., Narasu M.L., Singh O.V. (2008). The realm of penicillin G acylase in β-lactam antibiotics. Enzyme Microb. Technol..

[2] Fernandez-Lafuente R. (2009). Stabilization of multimeric enzymes: Strategies to prevent subunit dissociation. Enzyme Microb. Technol..

[3] Iyer P.V., Ananthanarayan L. (2008). Enzyme stability and stabilization-Aqueous and non-aqueous environment. Process Biochem..

[4] Kallenberg A.I., Van Rantwijk F., Sheldon R.A. (2005). Immobilization of penicillin G acylase: the key to optimum performance. Adv. Synth. Catal..

[5] Guisàn J.M., Alvaro G., Fernàndez-Lafuente R., Rosell C.M., Garcia J.L., Tagliani A. (1993). Stabilization of heterodimeric enzyme by multipoint covalent immobilization: Penicillin G acylase from *Kluyvera citrophila*. Biotechnol. Bioeng..

[6] Mateo C., Abiàn O., Fernàndez-Lorente G., Pedroche J., Fernàndez-Lafuente R., Guisàn J.M., Tam A., Daminati M. (2002). Epoxy Sepabeads: A novel epoxy support for stabilization of industrial enzymes via very intense multipoint covalent attachment. Biotechnol. Prog..

[7] Mateo C., Fernàndez-Lorente G., Abiàn O., Fernàndez-Lafuente R., Guisàn J.M. (2000). Multifunctional epoxy supports: a new tool to improve the covalent immobilization of proteins. The promotion of physical adsorptions of proteins on the supports before their covalent linkage. Biomacromolecules.

[8] Guisàn J.M. (1988). Aldehyde-agarose gels as activated supports for immobilization-stabilization of enzymes. Enzyme Microb. Technol..

[9] Alvaro G., Fernàndez-Lafuente R., Blanco R.M., Guisàn J.M. (1990). Immobilization-stabilization of penicillin G acylase from *Escherichia coli*. Appl. Biochem. Biotechnol..

[10] Mateo C., Abiàn O., Bernedo M., Cuenca E., Fuentes M., Fernàndez-Lorente G., Palomo J.M., Grazù V., Pessela B.C.C., Giacomini C. (2005). Some special features of glyoxyl supports to immobilize proteins. Enzyme Microb. Technol..

[11] Temporini C., Bonomi P., Serra I., Tagliani A., Bavaro T., Ubiali D., Massolini G., Terreni M. (2010). Characterization and study of the orientation of immobilized enzymes by tryptic digestion and HPLC-MS: design of an efficient catalyst for the synthesis of cephalosporins. Biomacromolecules.

[12] Mateo C., Palomo J.M., Fuentes M., Betancor L., Grazù V., Lòpez-Gallego F., Pessela B.C.C., Hidalgo A., Fernàndez-Lorente G., Fernàndez-Lafuente R. (2006). Glyoxyl agarose: a fully inert and hydrophilic support for immobilization and high stabilization of proteins. Enzyme Microb. Technol..

[13] Mateo C., Grazù V., Palomo J.M., Lopez-Gallego F., Fernàndez-Lafuente R., Guisàn J.M. (2007). Immobilization of enzymes on heterofunctional epoxy supports. Nat. Protoc..

[14] Serra I., Ubiali D., Cecchini D.A., Calleri E., Albertini A.M., Terreni M., Temporini C. (2013). Assessment of immobilized PGA orientation via the LC-MS analysis of tryptic digests of the wild type and its 3K-PGA mutant assists in the rational design of a high-performance biocatalyst. Anal. Bioanal. Chem..

[15] Penzol G., Armisen P., Fernàndez-Lafuente R., Rodes L., Guisàn J.M. (1998). Use of dextrans as long and hydrophilic spacer arms to improve the performance of immobilized proteins acting on macromolecules. Biotechnol. Bioeng..

[16] Fernàndez-Lafuente R., Rosell C.M., Rodriguez V., Guisàn J.M. (1995). Strategies for enzyme stabilization by intramolecular crosslinking with bifunctional reagents. Enzyme Microb. Technol..

[17] Adriano W.S., Filho E.H.C., Silva J.A., Giordano R.L.C., Goncalves L.R.B. (2005). Stabilization of penicillin G acylase by immobilization on glutaraldehyde-activated chitosan. Brazil. J. Chem. Eng..

[18] Fernàndez-Lorente G., Terreni M., Mateo C., Bastida A., Fernàndez-Lafuente R., Dalmases P., Huguet J., Guisàn J.M. (2001). Modulation of lipase properties in macro-aqueous systems by controlled enzyme immobilization: Enantioselective hydrolysis of a chiral ester by immobilized *Pseudomonas* lipase. Enzyme Microb. Technol..

[19] Fernàndez-Lafuente R., Rosell C.M., Caanan-Haden L., Rodes L., Guisàn J.M. (1999). Facile synthesis of artificial enzyme nano-environments via solid-phase chemistry of immobilized derivatives: dramatic stabilization of penicillin acylase *versus* organic solvents. Enzyme Microb. Technol..

[20] Jin X., Wu Q., Chen Q., Chen C.X., Lin X.F. (2008). Immobilization of penicillin G acylase on a composite carrier with a biocompatible microenvironment of chitosan. J. Chem. Technol. Biotechnol..

[21] Montes T., Grazù V., Manso I., Galan B., Lòpez-Gallego F., Gonzàlez R., Hermoso J.A., Garcia J.L., Guisàn J.M., Fernàndez-Lafuente R. (2007). Improved stabilization of genetically modified penicillin G acylase in the presence of organic cosolvents by co-immobilization of the enzyme with polyethyleneimine. Adv. Synth. Catal..

[22] Zhou C., Zhu S., Wu X., Jiang B., Cen T., Shen S. (2010). Post-immobilization of modified macromolecular reagents using assembled penicillin acylase for microenvironmental regulation of nanopores and enhancement of enzyme stability. Biotechnol. Bioproc. Eng..

[23] Fuentes M., Pessela B.C.C., Maquiese J.V., Ortiz C., Segura R.L., Palomo J.M., Abiàn O., Torres R., Mateo C., Fernàndez-Lafuente R. (2004). Reversible and strong immobilization of proteinsby ionic exchange on supports coated with sulfate-dextran. Biotechnol. Prog..

[24] Wilson L., Illanes A., Abiàn O., Pessela B.C.C., Fernàndez-Lafuente R., Guisàn J.M. (2004). Co-aggregation of penicillin G acylase and polyionic polymers: An easy methodology to prepare enzyme biocatalysts stable in organic media. Biomacromolecules.

[25] Rodrigues R.C., Ortiz C., Berenguer-Murcia A., Torres R., Fernández-Lafuente R. (2013). Modifying enzyme activity and selectivity by immobilization. Chem. Soc. Rev..

[26] Rodrigues R.C., Berenguer-Murcia A., Fernandez-Lafuente R. (2011). Coupling chemical modification and immobilization to improve the catalytic performance of enzymes. Adv. Synth. Catal..

[27] Kasche V. (1986). Mechanism and yields in enzyme catalysed equilibrium and kinetically controlled synthesis of β-lactam antibiotics, peptides and other condensation products. Enzyme Microb. Technol..

[28] Kasche V., Haufler U., Riechmann L. (1987). Equilibrium and kinetically controlled synthesis with enzymes: Semisynthesis of penicillins and peptides. Meth. Enzymol..

[29] Estruch I., Tagliani A.R., Guisàn J.M., Fernàndez-Lafuente R., Alcàntara A.R., Toma L., Terreni M. (2008). Immobilization of the acylase from *Escherichia coli* on glyoxyl-agarose gives efficient catalyst for the synthesis of cephalosporins. Enzyme Microb. Technol..

[30] Terreni M., Ubiali D., Bavaro T., Pregnolato M., Fernàndez-Lafuente R., Guisàn J.M. (2007). Enzymatic synthesis of cephalosporins. The immobilized acylase from *Arthrobacter viscosus*: A new useful biocatalyst. Appl. Microbiol. Biotechnol..

[31] Terreni M., Tchamkam J.G., Sarnataro U., Rocchietti S., Fernàndez-Lafuente R., Guisàn J.M. (2005). Influence of substrate structure on PGA-catalyzed acylations. Evaluation of different approaches for the enzymatic synthesis of cefonicid. Adv. Synth. Catal..

[32] Guisàn J.M., Penzol G., Armise P., Bastida A., Blanco R.M., Fernàndez-Lafuente R., Garcia-Giunceda E. (1997). Immobilization of Enzymes and Cells.

[33] Barbosa O., Torres R., Ortiz C., Berenguer-Murcia A., Rodrigues R.C., Fernandez-Lafuente R. (2013). Heterofunctional supports in enzyme immobilization: from traditional immobilization protocols to opportunities in tuning enzyme properties. Biomacromolecules.

[34] Serra I., Cecchini D.A., UbiaIi D., Manazza E.M., Albertini A.M., Terreni M. (2009). Coupling of site-directed mutagenesis and immobilization for the rational design of more efficient biocatalysts: The case of immobilized 3G3K PGA from *E. coli*. Eur. J. Org. Chem..

[35] Scaramozzino F., Estruch I., Rossolillo P., Terreni M., Albertini A.M. (2005). Improvement of catalytic properties of *Escherichia coli* penicillin G acylase immobilized on glyoxyl agarose by addition of a six-amino-acid tag. Appl. Environ. Microbiol..

[36] Migneault I., Dartiguenave C., Bertrand M.J., Waldron K.C. (2004). Glutaraldehyde: behaviour in aqueous solution, reaction with proteins, and application to enzyme crosslinking. Biotechniques.

[37] Boller T., Meier C., Menzler S. (2002). EUPERGIT oxirane acrylic beads: How to make enzymes fit for biocatalysis. Org. Process Res. Dev..

