# Supplementary material for: Modulation of the Microenvironment Surrounding the Active Site of Penicillin G Acylase Immobilized on Acrylic Carriers Improves the Enzymatic Synthesis of Cephalosporins
Source: Molecules. 2013 Nov 20;18(11):14349–65. doi: 10.3390/molecules181114349 (PMC6290566; doi:10.3390/molecules181114349)

Supplementary Materials

Figure S1. Percentage of conversion of *R*-(–)-mandelyl-7-ACA at increasing concentrations (5–50 mM) of 7-ACA.

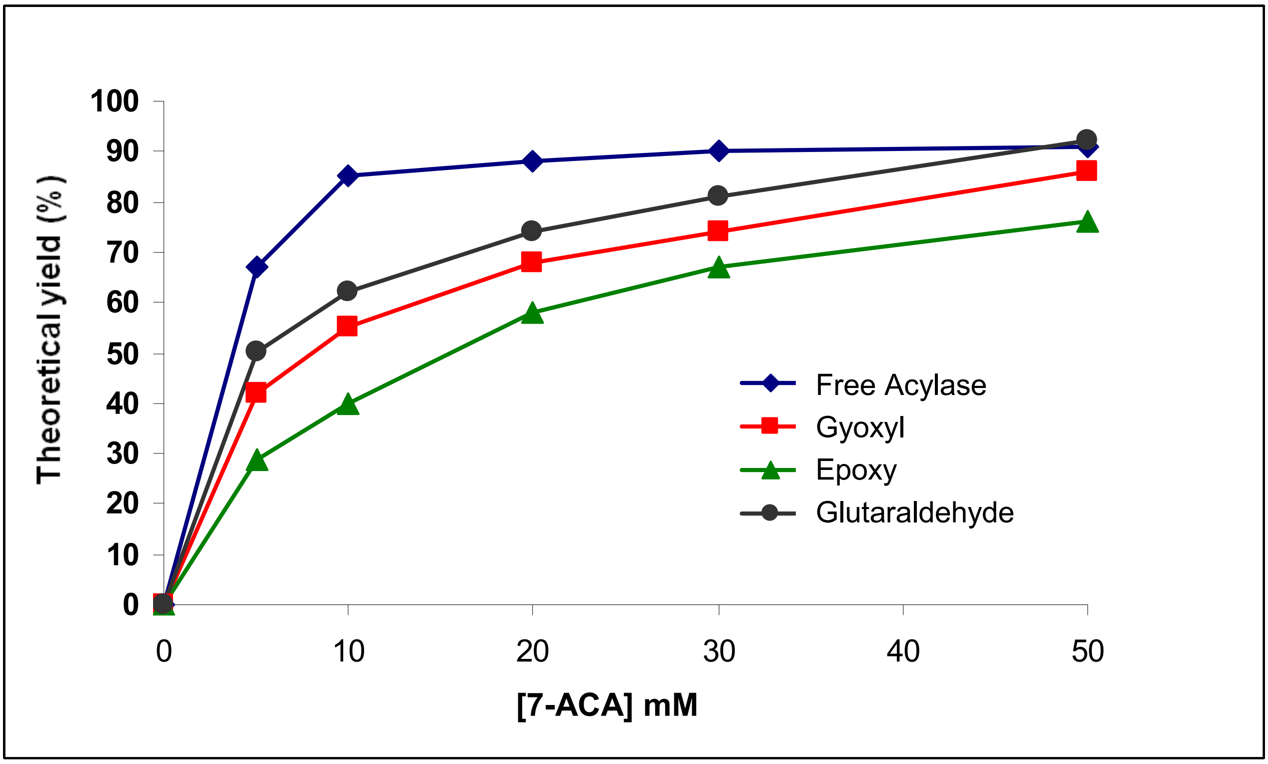

Reaction conditions: 4 °C, pH 6.5, *R*-(−)-methyl mandelic ester: 5 mM. *Legend*: non immobilized PGA (blue); PGA immobilized on glyoxyl agarose (red); PGA immobilized on epoxy acrylic supports (green); PGA immobilized on glutaraldehyde-activated acrylic support (black).

Figure S2. Stability of PGA immobilized on Eupergit® C before (blue) and after quenching with 1.5 M cysteine (red). Experimental conditions: 50 °C (panel A); MeOH 40% (v/v) (panel B).

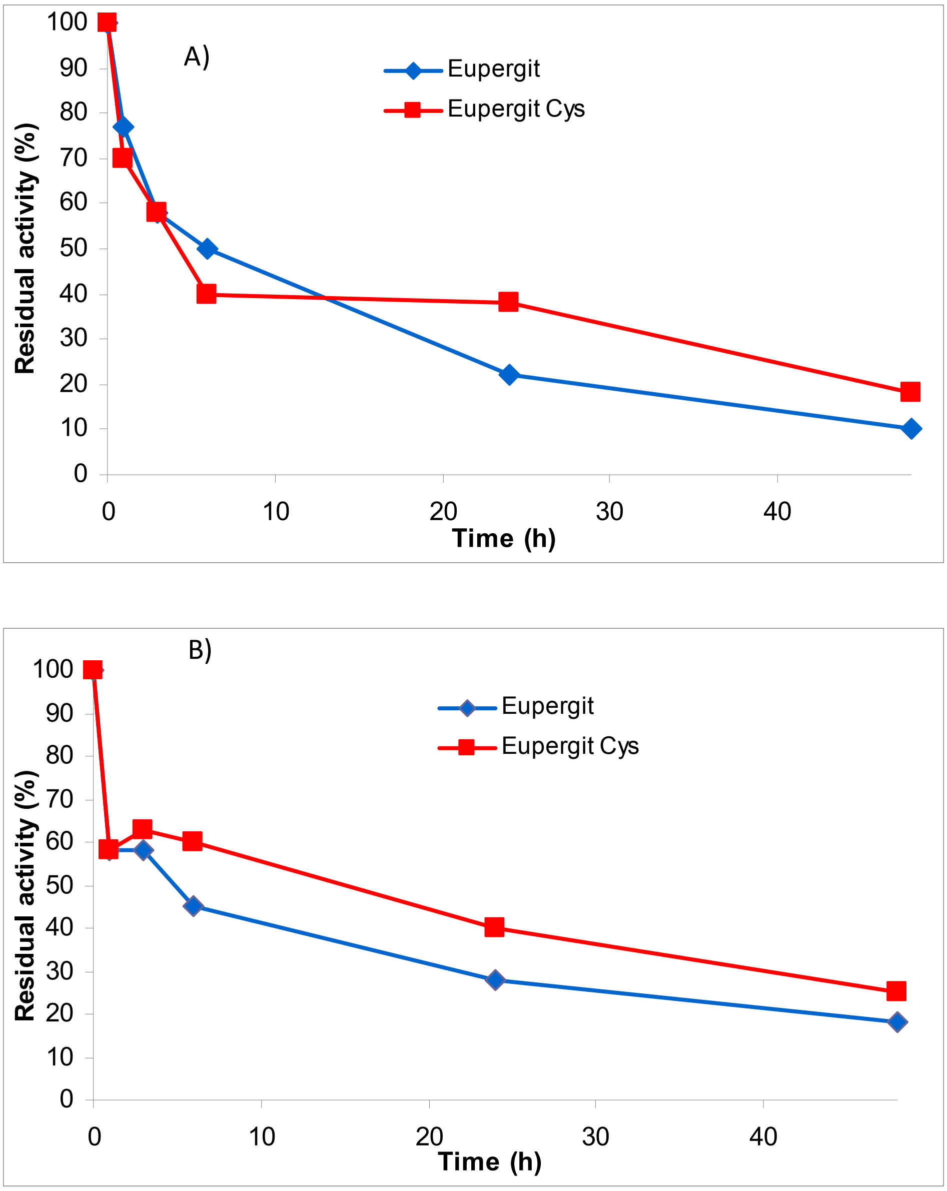

Supplement: Supplementary file 1 [file molecules-18-14349-s001.pdf]
